# Phylogenetic Analysis of *Francisella tularensis* Group A.II Isolates from 5 Patients with Tularemia, Arizona, USA, 2015–2017

**DOI:** 10.3201/eid2505.180363

**Published:** 2019-05

**Authors:** Dawn N. Birdsell, Hayley Yaglom, Edwin Rodriguez, David M. Engelthaler, Matthew Maurer, Marlene Gaither, Jacob Vinocur, Joli Weiss, Joel Terriquez, Kenneth Komatsu, Mary Ellen Ormsby, Marette Gebhardt, Catherine Solomon, Linus Nienstadt, Charles H.D. Williamson, Jason W. Sahl, Paul S. Keim, David M. Wagner

**Affiliations:** Northern Arizona University, Flagstaff, Arizona, USA (D.N. Birdsell, C.H.D. Williamson, J.W. Sahl, P.S. Keim, D.M. Wagner);; Arizona Department of Health Services, Phoenix, Arizona, USA (H. Yaglom, J. Weiss, K. Komatsu);; Navajo County Public Health Services District, Show Low, Arizona, USA (E. Rodriguez, C. Solomon);; Translational Genomics Research Institute, Flagstaff (D.M. Engelthaler);; Coconino County Public Health Services District, Flagstaff (M. Maurer, M. Gaither, M.E. Ormsby, M. Gebhardt);; Northern Arizona Healthcare, Flagstaff (J. Vinocur, J. Terriquez, L. Nienstadt)

**Keywords:** *Francisella tularensis*, tularemia, Arizona, group A.II, United States, bacteria, whole-genome sequencing, phylogenetic analysis, evolution

## Abstract

We examined 5 tularemia cases in Arizona, USA, during 2015–2017. All were caused by *Francisella tularensis* group A.II. Genetically similar isolates were found across large spatial and temporal distances, suggesting that group A.II strains are dispersed across long distances by wind and exhibit low replication rates in the environment.

*Francisella tularensis*, a Tier 1 select agent (*1*), has 3 subspecies: *tularensis* (type A), *holarctica* (type B), and *mediasiatica* (Appendix 1 Figure). In humans, disease is caused by type A and type B. Type B is found throughout the Northern Hemisphere, type A only in North America, and *mediasiatica* only in central Asia (*2*). Type A is divided into 2 distinct subgroups, A.I and A.II (Appendix 1 Figure), that have little geographic overlap (*3*,*4*). A.II is found primarily in the mountainous region of western North America (*3*,*4*) and A.I throughout the central eastern regions and along the West Coast (*3*–*5*). Observational human data and limited experimental mouse data suggest A.II is less virulent than A.I but potentially more virulent than type B (*6*,*7*). Here, we describe 5 patients in Arizona, USA, during 2015–2017 with cases of tularemia (1 fatal), all caused by A.II (Appendix 2).

## The Study

Case-patient 1 was a 57-year-old previously healthy man who sought treatment July 12, 2015, for chills and an acute onset of fever >40°C. Five days before symptom onset, while camping at the northern rim of Grand Canyon National Park, he noted a small wound at the lateral aspect of his left elbow consistent with an insect bite. Cellulitis with regional lymphadenopathy developed on his left forearm, extending to the left axillary region. After surgical irrigation, debridement of the wound (August 8), and oral doxycycline treatment upon discharge, the patient fully recovered.

Case-patient 2 was a 55-year-old previously healthy woman who sought treatment on July 20, 2015, for sore throat and an acute onset of fever >40°C. She reported no outdoor activity except being in a Coconino County park 4 days before symptom onset. Despite receiving treatment with amoxicillin, her fever persisted; she returned 4 days later with myalgia, fatigue, headaches, and emesis. Her therapy was switched to sulfamethoxazole/trimethoprim, amoxicillin/clavulanate, and ceftriaxone. A 2-day hospitalization revealed left axillary lymphadenopathy with associated cellulitis in her left chest wall and breast. Her fever resolved with intravenous ceftriaxone and gentamicin. She received oral doxycycline upon discharge and fully recovered.

Case-patient 3 was a 73-year-old Coconino County woman with previous health conditions. She sought treatment in the summer of 2016 and died several days later (Appendix 2 Table). Details about this case-patient are presented elsewhere (*8*).

Case-patient 4 was a 24-year-old previously healthy woman from Navajo County who sought treatment in November 2016. A cat bite was the suspected source of infection, but the cat was euthanized without testing. Severe swelling and lymphadenopathy developed at the site of the bite; the patient was treated with antimicrobial drugs and recovered.

Case-patient 5 was a 52-year-old man who resided and traveled between both Coconino County and Pinal County. He sought treatment for dizziness, nausea, chills, headache, and body aches in June 2017. He was initially treated with antipyretics but returned to the hospital 2 days later. At this visit, he received treatment with several antimicrobial drugs and recovered. The source of his infection is unknown.

All illnesses were classified as ulceroglandular tularemia except the one in case-patient 3, which was classified as respiratory tularemia. Recovered isolates from all 5 patients tested positive for *F. tularensis* group A.II by PCR (Appendix 2 Table).

Comparisons of whole-genome sequencing and geographic data (Appendix 1) for these 5 isolates and 9 other A.II isolates (Appendix 2 Table) revealed 2 patterns. First, the 5 cases in humans during 2015–2017 were caused by isolates from distinct clades (Figure, panel A). The isolates in case-patients 2 and 3, who probably acquired the infection in the same city, were assigned to 2 different major phylogenetic clades (A.II.2 and A.II.8), suggesting distinct clades co-occur locally in the environment, a finding similar to that observed with type B and group A.I (*9*,*10*). Second, some closely related isolates were distant in geographic and temporal space (Figure, panels A, B). Isolates from case-patients 3 and 4 are highly similar, differing by just 1 single-nucleotide polymorphism (SNP) across the core genome, despite being isolated >150 km and 5 months apart; they also differ by just 3–4 SNPs from a case that occurred in another location 9 years previous in 2007 (no. 8; Figure). Likewise, isolates from case-patients 2 and 5 differ by just 4 SNPs, despite being isolated from distant locations 2 years apart.

**Figure:**
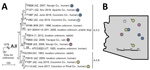
Phylogeny and geographic distribution of *Francisella tularensis* isolates, Arizona, 2005–2017. A) Maximum-parsimony tree of 14 *F. tularensis* subsp. *tularensis* A.II isolates from humans and other mammals constructed by using single-nucleotide polymorphisms (SNPs) discovered by whole-genome sequencing. The tree is rooted on A.I strain Schu S4. Scale bar indicates number of SNPs. Numbers along branches also indicate the number of SNPs the branches represent. Closely related isolates are indicated with circles of the same color (also indicated in panel B). Numbers within circles correspond to the identification numbers in Appendix 2 Table. B) Known or suspected geographic origins of tularemia cases in Arizona. *Case 5 is represented twice to reflect the 2 possible geographic sources of this infection. Co., county.

The geographic pattern suggests *F. tularensis* group A.II might be capable of long-distance dispersal, perhaps by wind, as has been suggested for type B (*9*,*11*). The temporal pattern, which also has been observed for type B (*9*,*11*), is consistent with a low evolutionary rate for A.II strains in the environment. This pattern suggests group A.II strains may persist in the environment in a dormant state, such as the viable but nonculturable state that has been described for type B (*12*).

Consistent with a low evolutionary rate in the environment, groups A.I and A.II appear to be highly monomorphic and have much less genetic variation than type B (Appendix 1 Figure). Type B was previously described as being less genetically diverse than type A as a whole when groups A.I and A.II were considered together (*2*). However, A.I and A.II are separated by large SNP distances with no intermediate lineages (Appendix 1 Figure), verifying these groups as highly distinct and warranting their analysis as separate groups.

In this study, just 309 SNPs were discovered among 14 A.II isolates separated by considerable geographic (maximum >1,000 km) and temporal (maximum 96 years) distances (Figure, panel A). In a previous study (*13*), just 295 SNPs were discovered among 14 A.I isolates separated by similar temporal distances (maximum 65 years) and an even greater geographic distance (maximum >2,800 km). In contrast, type B exhibits much more diversity across smaller geographic and temporal scales. For example, 735 SNP differences were found in an analysis of 10 isolates from a respiratory tularemia outbreak in Sweden (*9*), even though the temporal (maximum 1 year) and geographic (maximum ≈201 km) distances among these isolates were much smaller. The patterns observed with group A.II isolates suggest that, as has been suggested for type B (*9*,*11*), both group A.I and A.II strains might also persist long term in the environment in a dormant state where replication is nonexistent or greatly arrested.

A.II appears to be the main and perhaps only group of *F. tularensis* present in the environment in Arizona, although group A.I and type B are known to be present in neighboring states (*14*). However, all available archival isolates from human and wildlife sources in Arizona (Appendix 2 Table) were assigned to the A.II group (Appendix 1), consistent with other reports, indicating the presence of only group A.II from animal and human sources from Arizona (*3*,*4*). In 2000, type B isolates were obtained from captive animals in an Arizona zoo, but these infections were suspected to be imported rather than locally acquired (*2*).

## Conclusions

In summary, we report 5 cases of tularemia in humans (including 1 fatality) that occurred in Arizona during 2015–2017, and all were caused by A.II isolates. Phylogeographic patterns suggest *F. tularensis* A.II strains might persist in the environment in a dormant state and be dispersed long distances, perhaps by wind.

**Appendix 1.** Methods used for studying 5 cases of *Francisella tularensis* group A.II infection in humans, Arizona, USA, 2015–2017, and phylogenetic relatedness of 3 *F. tularensis* subspecies.

**Appendix 2.** Information on 5 *Francisella tularensis* group A.II cases in humans, Arizona, USA, 2015–2017, and 9 other *F. tularensis* group A.II cases across the United States.
