# Supplementary material for: Phylogenetic Analysis of Francisella tularensis Group A.II Isolates from 5 Patients with Tularemia, Arizona, USA, 2015–2017
Source: Emerg Infect Dis. 2019 May;25(5):944–6. doi: 10.3201/eid2505.180363 (PMC6478195; doi:10.3201/eid2505.180363)
Supplement: Supplementary file 1 — Appendix 1. Methods used for studying 5 cases of Francisella tularensis group A.II infection in humans, Arizona, USA, 2015–2017, and phylogenetic relatedness of 3 F. tularensis subspecies. [file 18-0363-Techapp-s1.pdf]

Article DOI: https://doi.org/10.3201/eid2505.180363

Phylogenetic Analysis of *Francisella tularensis* Group A.II Isolates from 5 Patients with Tularemia, Arizona, USA, 2015–2017

Appendix

Francisella tularensis Culture, DNA Extraction, and Assignment to Major Genetic Groups

For case 4 (Northern Arizona University identification no. F1071), *Francisella tularensis* was obtained from the wound site 10 days after the initial onset of symptoms and isolated by culture at a commercial laboratory. The sample was confirmed positive at the Arizona State Public Health Laboratory (Phoenix, Arizona, USA), which provided Northern Arizona University (Flagstaff, Arizona, USA) staff with DNA extracts of the cultured isolate for analysis. Information on the procedures used to confirm *F. tularensis* in each case is presented in Appendix 2 Table (https://wwwnc.cdc.gov/EID/article/25/5/18-0363-App2.xlsx).

Isolates were first cultured in the hospitals where patients received medical attention; isolates were sent to Northern Arizona University for additional analysis. We cultured on Cysteine Heart Agar (Remel, https://www.thermofisher.com) for 72 hours at 37°C. DNA was extracted by using QIAGEN kits (https://www.qiagen.com). DNA extracts of *F. tularensis* isolates were genetically typed into phylogenetic groups by using published assays (*1*).

DNA Sequencing

We sequenced the DNA extracts of 10 isolates (the 5 isolates we obtained from humans in Arizona and 5 archival isolates from humans, environmental sources, or sources of unknown origin; Appendix 2 Table). DNA library preparations for whole-genome sequencing were performed by using the KAPA Low Throughput Library Preparation Kit with SPRI Solution and

Standard PCR Library Amplification/Illumina series (KK8232; KAPA Biosystems, https://www.kapabiosystems.com) with modifications. The adapters and 8-bp index oligos were based on Kozarewa and Turner (*2*) and purchased from Integrated DNA Technologies (https://www.idtdna.com). The quality and quantity of genomic DNA was evaluated by 0.7% agarose gel analysis. We fragmented ≈1 ug of DNA per sample with a SonicMan (Matrical Technologies, https://www.matrical.in) using the following parameters: 75.0-s prechill, 16 cycles, 10.0-s sonication, 100% power, 75.0-s lid chill, 10.0-s plate chill, and 75.0-s postchill. The sonicated DNA was size selected to target 600–650 bp by performing fragment separation with Agencourt AMPure XP beads (A63882; Beckman Coulter, https://www.beckmancoulter.com); the fragmented DNA was eluted into 42.5 μL of elution buffer.

During the enzymatic steps, half the enzyme volume was used with the full volume of buffer as described in the KAPA's library preparation protocol. For library preparation, 3 separate reactions were carried out for each sample. In the first reaction (end repair), 2.5 μL of enzyme and 5 μL of buffer were used, followed by a 1.6× AMPure XP bead cleanup and elution in 43.5 μL of elution buffer. In the second reaction (A-tailing), 1.5 μL of enzyme and 5 μL of buffer were used, followed by a 1.6× AMPure XP bead cleanup and elution in 36.5 μL of elution buffer. In the third reaction (quick ligation), we used 2.5 μL of enzyme, 10 μL of buffer, and 1 μL of 10 μM adaptor oligo mix (*2*), followed by a 0.9× AMPure XP bead cleanup and elution in 30 μL of elution buffer. The PCR was optimized to improve yield and genome coverage.

We combined 2 μL of DNA, 2 μL of each 10 μM indexing primer, 25 μL of KAPA 2× HIFI PCR Master Mix (KAPA Biosystems), and 19 μL of molecular grade water and used the following PCR parameters: 2 min at 98°C (initial denaturation); 8 cycles of 30 s at 98°C, 20 s at 65°C, and 30 s at 72°C (amplification); and 5 min at 72°C (final extension). The libraries were purified with a 0.9× AMPure XP bead cleanup and eluted into 50.0 μL of elution buffer. The final libraries were quantified by using the KAPA ABI Prism Library Quantification Kit (KK4835; KAPA Biosystems) and pooled together at equimolar concentrations.

The pool was quantified by using the KAPA ABI Prism Library Quantification Kit, and the quality of the pool was assessed with the Agilent DNA 1000 Kit (5067-1504; Agilent

Technologies, https://www.agilent.com) on the Bioanalyzer DNA 1000 Chip (Agilent Technologies). The samples, spiked with 1% PhiX, were sequenced on an Illumina MiSeq instrument by using the 600-cycle MiSeq Reagent Kit v3 (MS-102-3003; Illumina, https://www.illumina.com) with the standard Illumina procedure. The appropriate sequencing primers were added to the MiSeq kit as described (*3*). Sequence data was submitted to NCBI under BioProject PRJNA398413 (Sequence Read Archive accession nos. SRR5937764–SRR5937772, SRR6205344; Appendix 2 Table).

Single-Nucleotide Polymorphism Discovery and Phylogeny

Core genome single-nucleotide polymorphisms (SNPs) were identified among *F. tularensis* genomes with NASP (*4*). Reads were aligned to the reference assembly (GenBank accession no. NC_006570.2) with Bowtie 2 (used for SNP identification) (*5*). If reads were not available for a genome, paired Illumina reads were simulated for the genome with ART (*6*). SNPs were identified with the UnifiedGenotyper method in Genome Analysis Toolkit (*7*,*8*). SNPs were removed from the matrix if the read depth was <10× or the allele frequency proportion was <90%. SNPs identified in duplicated regions of the reference assembly with NUCmer self-alignments (*9*,*10*) were filtered from the SNP matrix. A total of 1,308 SNPs were called from a 1,140,352-bp core genome alignment (NASP quality_breadth). Two SNPs were phylogenetically conflicting. A maximum parsimony tree was inferred on high-quality, core genome SNPs with MEGA 6.0 (*11*). SCHU S4 (GenBank accession no. NC_006570.2) was the outgroup for the phylogenetic analyses.

References

1. Birdsell DN, Vogler AJ, Buchhagen J, Clare A, Kaufman E, Naumann A, et al. TaqMan real-time PCR assays for single-nucleotide polymorphisms which identify *Francisella tularensis* and its subspecies and subpopulations. PLoS One. 2014;9:e107964. PubMed http://dx.doi.org/10.1371/journal.pone.0107964

2. Kozarewa I, Turner DJ. Amplification-free library preparation for paired-end Illumina sequencing. Methods Mol Biol. 2011;733:257–66. PubMed http://dx.doi.org/10.1007/978-1-61779-089-8_18

3. Kozarewa I, Turner DJ. 96-plex molecular barcoding for the Illumina Genome Analyzer. Methods Mol Biol. 2011;733:279–98. PubMed http://dx.doi.org/10.1007/978-1-61779-089-8_20

4. Sahl JW, Lemmer D, Travis J, Schupp JM, Gillece JD, Aziz M, et al. NASP: an accurate, rapid method for the identification of SNPs in WGS datasets that supports flexible input and output formats. Microb Genom. 2016;2:e000074. PubMed http://dx.doi.org/10.1099/mgen.0.000074

5. Langmead B, Salzberg SL. Fast gapped-read alignment with Bowtie 2. Nat Methods. 2012;9:357–9. PubMed http://dx.doi.org/10.1038/nmeth.1923

6. Huang W, Li L, Myers JR, Marth GT. ART: a next-generation sequencing read simulator. Bioinformatics. 2012;28:593–4. PubMed http://dx.doi.org/10.1093/bioinformatics/btr708

7. DePristo MA, Banks E, Poplin R, Garimella KV, Maguire JR, Hartl C, et al. A framework for variation discovery and genotyping using next-generation DNA sequencing data. Nat Genet. 2011;43:491–8. PubMed http://dx.doi.org/10.1038/ng.806

8. McKenna A, Hanna M, Banks E, Sivachenko A, Cibulskis K, Kernytsky A, et al. The Genome Analysis Toolkit: a MapReduce framework for analyzing next-generation DNA sequencing data. Genome Res. 2010;20:1297–303. PubMed http://dx.doi.org/10.1101/gr.107524.110

9. Delcher AL, Phillippy A, Carlton J, Salzberg SL. Fast algorithms for large-scale genome alignment and comparison. Nucleic Acids Res. 2002;30:2478–83. PubMed http://dx.doi.org/10.1093/nar/30.11.2478

10. Kurtz S, Phillippy A, Delcher AL, Smoot M, Shumway M, Antonescu C, et al. Versatile and open software for comparing large genomes. Genome Biol. 2004;5:R12. PubMed http://dx.doi.org/10.1186/gb-2004-5-2-r12

11. Tamura K, Stecher G, Peterson D, Filipski A, Kumar S. MEGA6: Molecular Evolutionary Genetics Analysis version 6.0. Mol Biol Evol. 2013;30:2725–9. PubMed http://dx.doi.org/10.1093/molbev/mst197

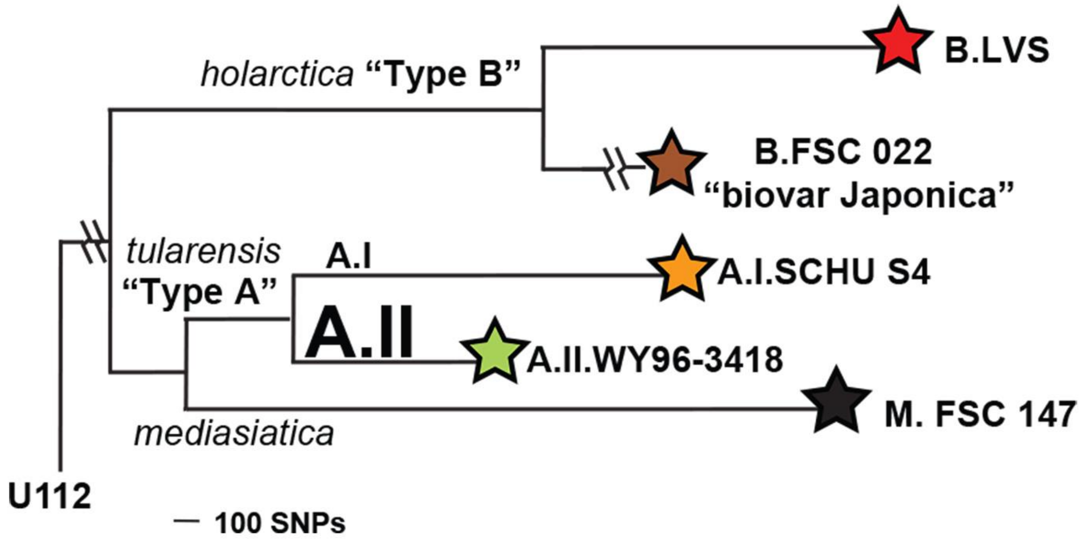

Appendix Figure. Phylogenetic tree indicating the major genetic groups and subgroups within *Francisella tularensis.* Colored stars indicate representative whole-genome sequences from these different groups, which were used to construct this phylogeny. SNP, single-nucleotide polymorphism.
